# Real-time deflection and friction force imaging by bimorph-based resonance-type high-speed scanning force microscopy in the contact mode

**DOI:** 10.1186/1556-276X-9-665

**Published:** 2014-12-10

**Authors:** Wei Cai, Haiyun Fan, Jianyong Zhao, Guangyi Shang

**Affiliations:** 1 Department of Applied Physics, Beihang University, Beijing 100191, People’s Republic of China; 2 Key Laboratory of Micro-nano Measurement-Manipulation and Physics (Ministry of Education), Beihang University, Beijing 100191, People’s Republic of China

**Keywords:** Atomic force microscopy, High-speed atomic force microscopy, Friction force microscopy, Resonance-type bimorph scanner

## Abstract

We report herein an alternative high-speed scanning force microscopy method in the contact mode based on a resonance-type piezoelectric bimorph scanner. The experimental setup, the modified optical beam deflection scheme suitable for smaller cantilevers, and a high-speed control program for simultaneous data capture are described in detail. The feature of the method is that the deflection and friction force images of the sample surface can be obtained simultaneously in real time. Images of various samples (e.g., a test grating, a thin gold film, and fluorine-doped tin oxide-coated glass slides) are acquired successfully. The imaging rate is 25 frames per second, and the average scan speed reaches a value of approximately 2.5 cm/s. The method combines the advantages of both observing the dynamic processes of the sample surface and monitoring the frictional properties on the nanometer scale.

**PACS:**

07.79.Lh; 07.79.Sp; 68.37.Ps

## Background

Experimental methods for nanotribology studies by means of atomic force microscopy (AFM) have been developed rapidly during the past decade [1-3]. AFM or friction force microscopy (FFM) [4], which is known as one of AFM's derivative technologies, has been widely used for detecting frictional properties of sample surfaces with extraordinary resolution [5-8]. In FFM, a sharp tip that is at the end of a cantilever contacts the sample surface slightly. During scanning, lateral/friction force would twist the cantilever from its equilibrium position [9]. By using the optical beam deflection (OBD) technique, the normal and the lateral forces detected by the tip can be measured simultaneously [10,11]. With these characteristics of FFM, various novel phenomena are accessible to researchers, such as the observation of the atomic stick-slip friction [12], measurement of a single-asperity contact friction [9], and research of the velocity-dependent friction [13]. Despite these advantages, the primary disadvantage of the traditional FFM technique is the slow scan velocity in friction phenomena research. Usually, several minutes are required to get an image with frictional information. The corresponding scan velocity is restricted in the range of micrometers per second. It is much lower than the sliding velocity in the practical use, e.g., applications in storage mediums or MEMS devices [14-16].

To solve this problem, on one hand, some researchers modified the traditional instrument by adding extra components to acquire the rapid motion of the sample. For example, Tocha et al. added a shear piezo element on the existing stand-alone AFM; the scan velocity can get up to several millimeters per second [17]. Tao and Bhushan used a custom-calibrated piezo-actuator stage with a scan velocity of up to 200 mm/s [14]. Zhang et al. employed quartz crystal resonators with 3-MHz resonance frequency; the scan velocity reached up to several meters per second [18]. All these proposed methods break the speed limits of traditional FFM in friction research. However, these devices aforementioned have almost no ability to provide high-speed friction force images simultaneously. On the other hand, the advent of high-speed AFM (HSAFM) has created new opportunities for the observation of dynamic processes on the sample surface at nanoscale [19-24]. The scan velocity has been increased by developing high-speed scanners [19,25-30], smaller cantilevers [19,31,32], fast vertical feedback control algorithms [33,34], as well as cantilever excitation and modulation methods [35,36]. Comprehensive descriptions of techniques used in HSAFM can be found in several recently published reviews [23,24,37]. For friction research by HSAFM, Schitter and Stemmer improved a piezo tube-based AFM and applied a model-based open-loop control on it. Friction force images of aluminum triangles were obtained with a line scan rate at a moderate value of 122 Hz [38]. Lee et al. reported the study of surface wear problem in real time by means of HSAFM [39,40]. Hydrophobic tips and mineral oil were used to make a lubricious surface to decrease the wear. Topographic images were given, but high-speed friction force images were not provided directly.

In this paper, we present an alternative high-speed scanning force microscopy (HSSFM) method based on a resonance-type piezoelectric bimorph scanner, which has the ability to provide real-time deflection and friction force images in the contact mode simultaneously. The experimental setup, the modified OBD scheme for smaller cantilevers, control, and high-speed data acquisition (DAQ) program are described in detail. Images of a test grating, a thin gold film, and fluorine-doped tin oxide (FTO) glass slides are demonstrated. The imaging rate is 25 frames per second (FPS), and the average scan speed (*v*_a_) can attain a value of approximately 2.5 cm/s in experiments. These results show that the method can be used not only for the observation of the dynamic processes of the sample surface but also for real-time monitoring differences in frictional properties of the surface with nanometer-scale resolution. We believe that the HSSFM method would have great potential applications for real-time surface inspections, dynamic observation of changes in frictional properties, and nanotribology studies.

## Methods

**Figure 1:**
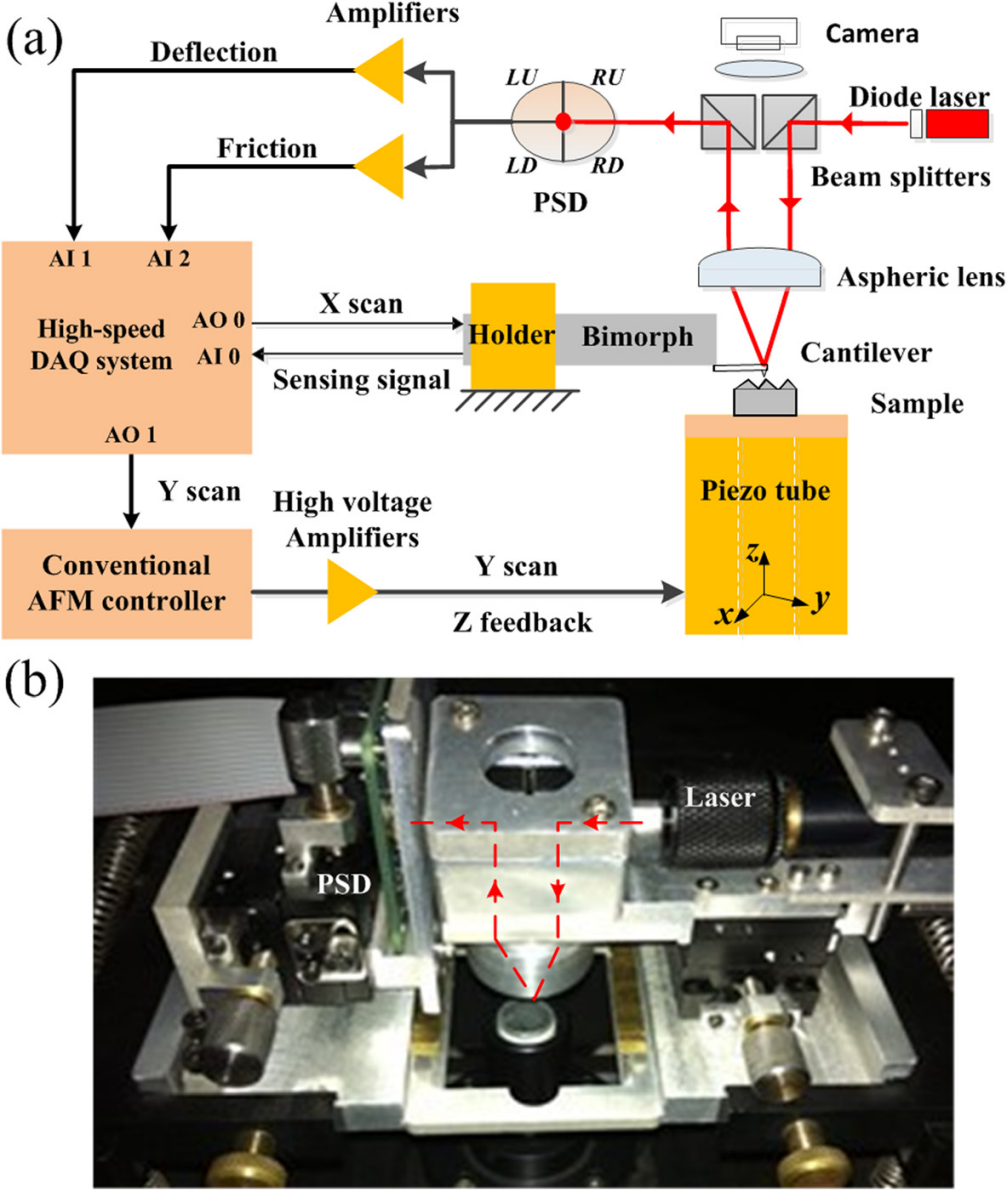
**Experimental setup. (a)** Schematic illustration of our experimental setup based on a resonance-type bimorph scanner for high-speed topographic and friction force microscopy. **(b)** Photograph of the setup showing the modified optical beam deflection scheme for smaller cantilevers. The dash line in the photograph is the working light path.

The schematic illustration of our homemade HSSFM experimental setup is shown in Figure 1a. During scanning, the tip-sample interaction forces would cause deflection and torsion of the cantilever. The small deformation of the cantilever would change the direction of the reflected laser beam, resulting in different position of the laser spot on the position-sensitive detector (PSD). If the signals from four cells of the PSD are denoted by *I*_LU_, *I*_LD_, *I*_RU_, and *I*_RD_, the total signal of the PSD can be written as *I*_sum_ = *I*_LU_ + *I*_RU_ + *I*_LD_ + *I*_RD_. Thus, the measured signals for imaging will be *I*_d_ = ((*I*_LU_ + *I*_RU_) - (*I*_LD_ + *I*_RD_))/*I*_sum_ and *I*_f_ = ((*I*_LU_ + *I*_LD_) - (*I*_RU_ + *I*_RD_))/*I*_sum_, which are proportional to the cantilever deflection and friction/torsion, respectively. The deflection signal *I*_d_ and the friction signal *I*_f_ are fed to two analog input (AI) channels of the high-speed DAQ system (S-6115, National Instruments Corp., Austin, TX, USA) for simultaneous high-speed deflection and friction force imaging.

In our experimental setup, fast scan (*x*-axis) was realized by using a resonance-type piezoelectric bimorph scanner [41,42]. Traditional piezo tube scanner provides slow scan (*y*-axis) and motion in *z*-axis for keeping an average height to the sample surface while imaging in high speed [20,24,25]. Two analog output (AO) converters in the DAQ system were employed for synchronized scan signal generation. Fast-scan (sine waveform) signal was connected to one AO channel directly, the voltage range was ±10 V. Slow-scan (triangle waveform) signal was generated from the other AO channel and then was amplified up to ±150 V. The conventional AFM controller provides the functions of coarse approach control for the tip and high-voltage amplifiers for the tube scanner.

The sensing signal from the bimorph scanner was connected to one AI channel, which could be used in the real-time image distortion correction [43]. Scan control and data acquisition processes were set to run synchronously in a homemade program, which was the key issue to realize real-time imaging. In order to improve the program efficiency, the data collected by the DAQ system was added to a queue and then read by the display module for imaging. The real-time processes, such as the image distortion correction of both deflection and friction channels, were set up to run in parallel.

Small cantilevers (BL-AC40TS-C2, Olympus Corp. Tokyo, Japan) were chosen in our experiments. The dimension of the cantilever is 38 μm × 16 μm × 0.2 μm. The resonance frequency is 110 kHz and the force constant is 0.1 N/m. Since the reflecting area on the back of the cantilever is very small, the OBD detection scheme in our experimental setup has been modified to meet the requirements for smaller cantilevers. The schematic illustration and the light path are also shown in Figure 1a. The unique feature of this modified scheme is the use of a large-diameter aspheric lens to reduce the size of the focused laser spot effectively. Two identical beam splitters were installed symmetrically on top of the aspheric lens. The laser diode module and the PSD were separately placed in the two sides of the beam splitters in a symmetric position with respect to the vertical central axis, which is beneficial to obtain a compact AFM head unit and for easy adjustment of the light path. The collimated laser beam generated from the diode laser module was reflected by one beam splitter and then focused by the aspheric lens at an off-axis position. The light reflected by the back of the cantilever passed through the same aspheric lens, and finally it was reflected into the PSD by the other beam splitter. A noticeable misalignment of the laser beam path was not found while the tip scanned the sample surface because the maximum scan range was limited by the software to be approximately 10 μm in our present experiments. This value was smaller than the size of the laser spot focused on the cantilever [42]. The cantilever can be monitored through an optical microscope above the beam splitters, which was helpful for the laser beam alignment.Figure 1b is a photograph of our HSSFM setup, where the dash line in the figure is the working light path. The aspheric lens is mechanically fixed by using a metal tube above the AFM cantilever. The position of the metal tube can be manually adjusted for focusing the laser on the back of the cantilever. The beam splitters were installed in a metal frame arranged above the aspheric lens. The optical microscope (not shown in this figure) for the laser beam alignment was mounted over the splitters and its base was fixed on a two-dimensional translation stage. The position of the laser diode module in the horizontal plane and the tilt angle can be adjusted, and the position of the PSD can also be finely adjusted in the vertical plane.

## Results and discussion

**Figure 2:**
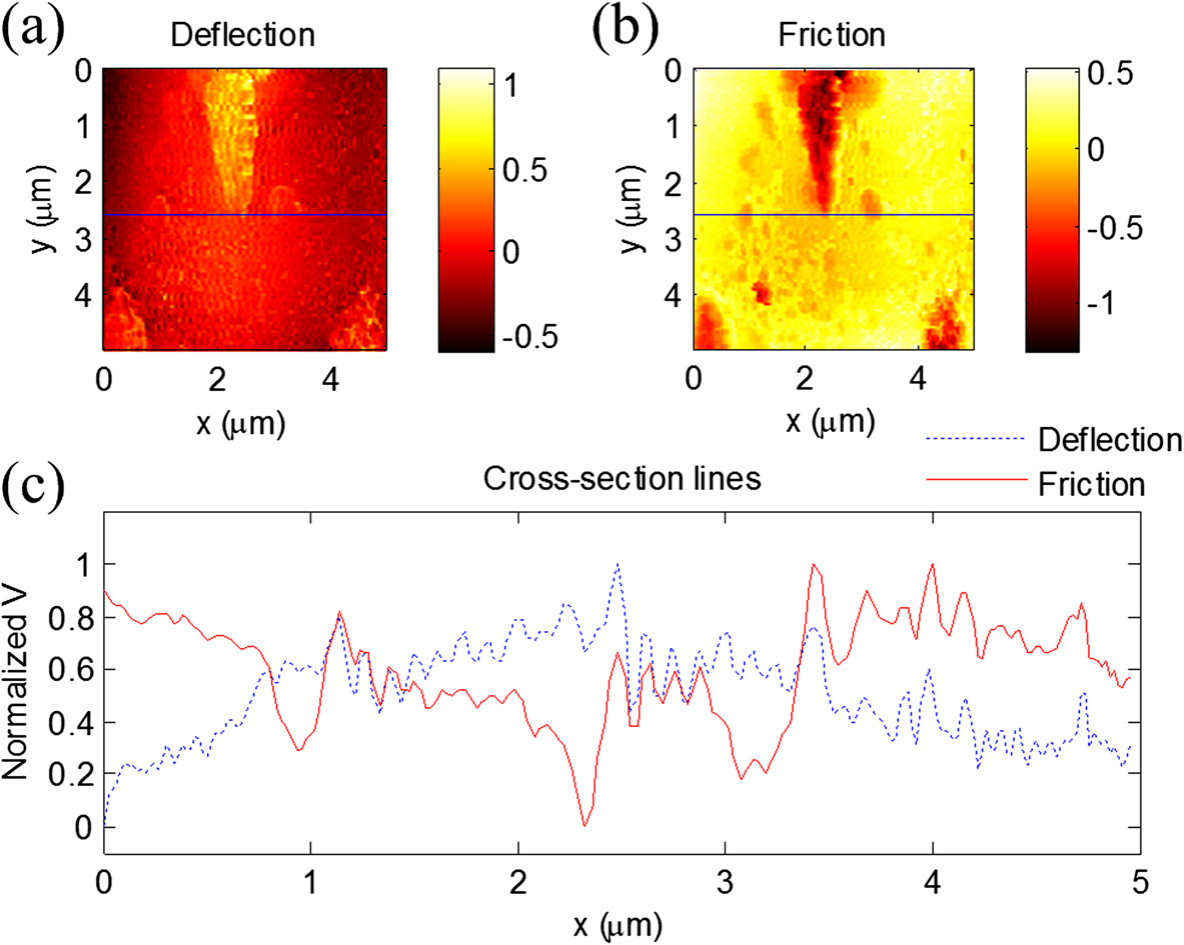
**HSSFM images of the test grating. (a)** Deflection and **(b)** friction force images of SNG01 test grating taken with our HSSFM. **(c)** Cross-section lines performed as shown in **(a)** and **(b)**.

**Figure 3:**
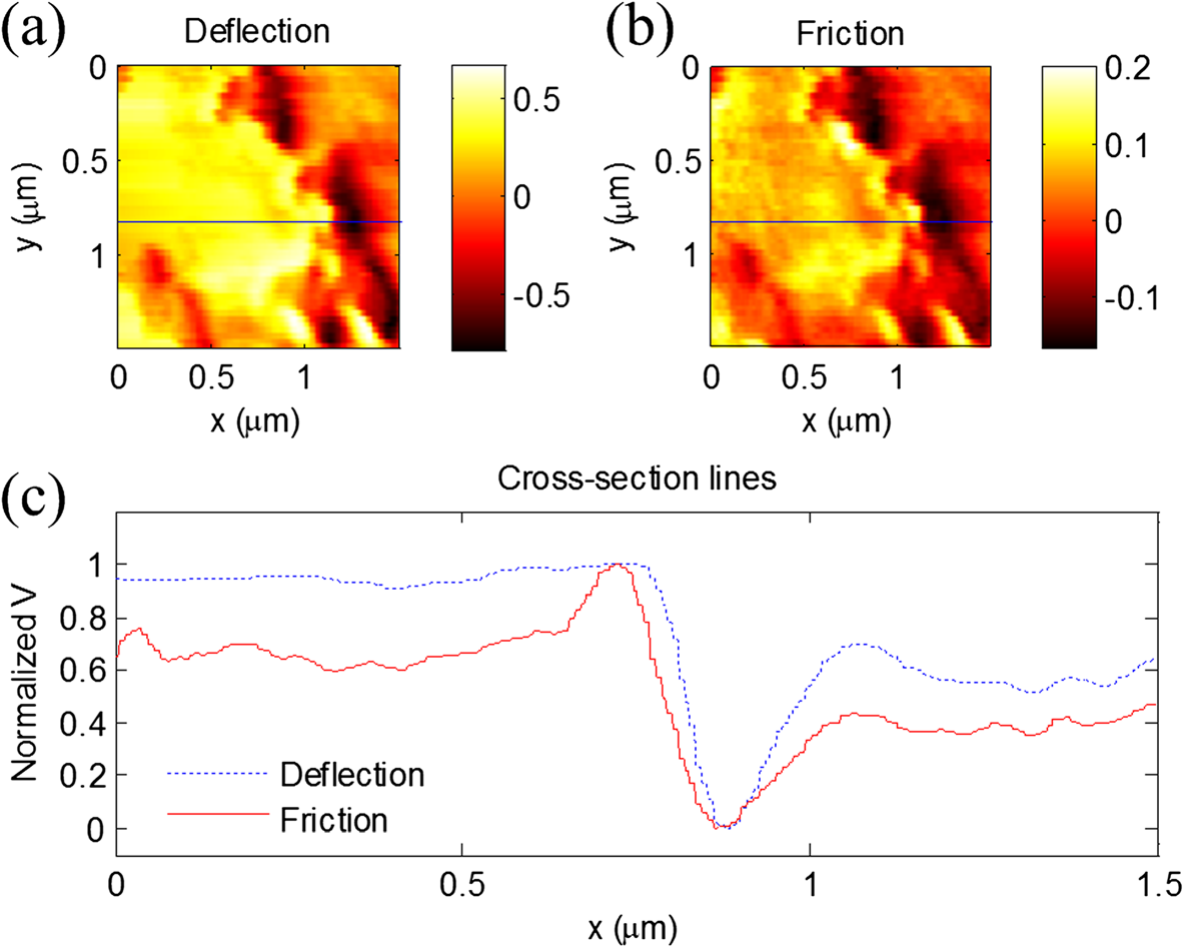
**HSSFM images of the gold film. (a)** Deflection and **(b)** friction force images of the gold film sample taken by our HSSFM. **(c)** Cross-section lines performed as shown in **(a)** and **(b)**. The line scan rate was set at approximately 2.5 kHz. These images were also acquired simultaneously at 25 FPS, and the scan size was approximately 1.5 μm × 1.5 μm.

To demonstrate the ability for high-speed topographic and friction force imaging, we firstly used the SNG01 grating (NT-MDT, Moscow, Russia) as the test sample. The sample, consisting of approximately 20-nm-thick vanadium rhombs on a quartz substrate, has two different materials on the surface. From the deflection image, as shown in Figure 2a, rhomb-shaped areas can be found on the surface. In the friction force image, as shown in Figure 2b, two different materials on the surface can be distinguished based on the contrast of the friction signal, since the frictional properties of the quartz and the vanadium area are obviously different. In fact, from the cross sections in the same position of images, it can be seen that compared with the deflection signal, the friction signal provides different contrast. In addition, some small particles on the substrate can be found in the friction force image, which are also due to the different fictional properties. The line scan rate was set at approximately 2.5 kHz. These images were acquired simultaneously at 25 FPS, and the scan size was approximately 5 μm × 5 μm. The *v*_a_ can be calculated by *v*_a_ = 2*f*_0_*L*, where *f*_0_ is the line scan rate and *L* is the scan size. So, the average scan speed in this experiment is approximately 2.5 cm/s. Thus, we can conclude that not only the topography but also the fictional properties of materials can be detected in real time with our HSSFM method.Secondly, a gold film prepared by ion sputtering on the glass substrate was chosen as another test sample. In the central part of the deflection image, as shown in Figure 3a, the topography of the gold film shows a flat area, i.e., the deflection signal shows almost no changes, which can be seen from the profile of the image in Figure 3c. However, more details can be obtained from the friction force images in the corresponding area and the profile line, as shown in Figure 3b,c, respectively. The results show that although there is only one kind of material on the surface, the preparation process might cause the local differences in frictional properties.

**Figure 4:**
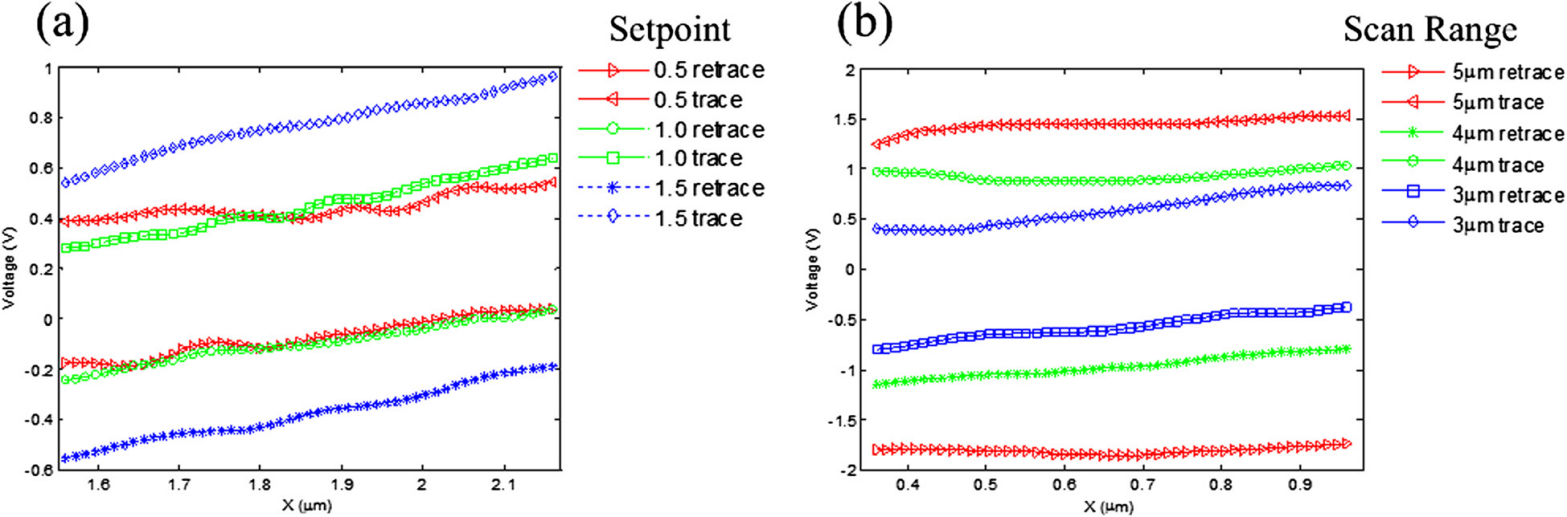
**Friction force signals. (a)** Friction force signals acquired on the surface of monocrystalline silicon both retrace (backward scan) and trace (forward scan) curves under different normal forces. **(b)** Friction force signals acquired on a surface of monocrystalline silicon both retrace and trace curves under different scan speeds.

**Figure 5:**
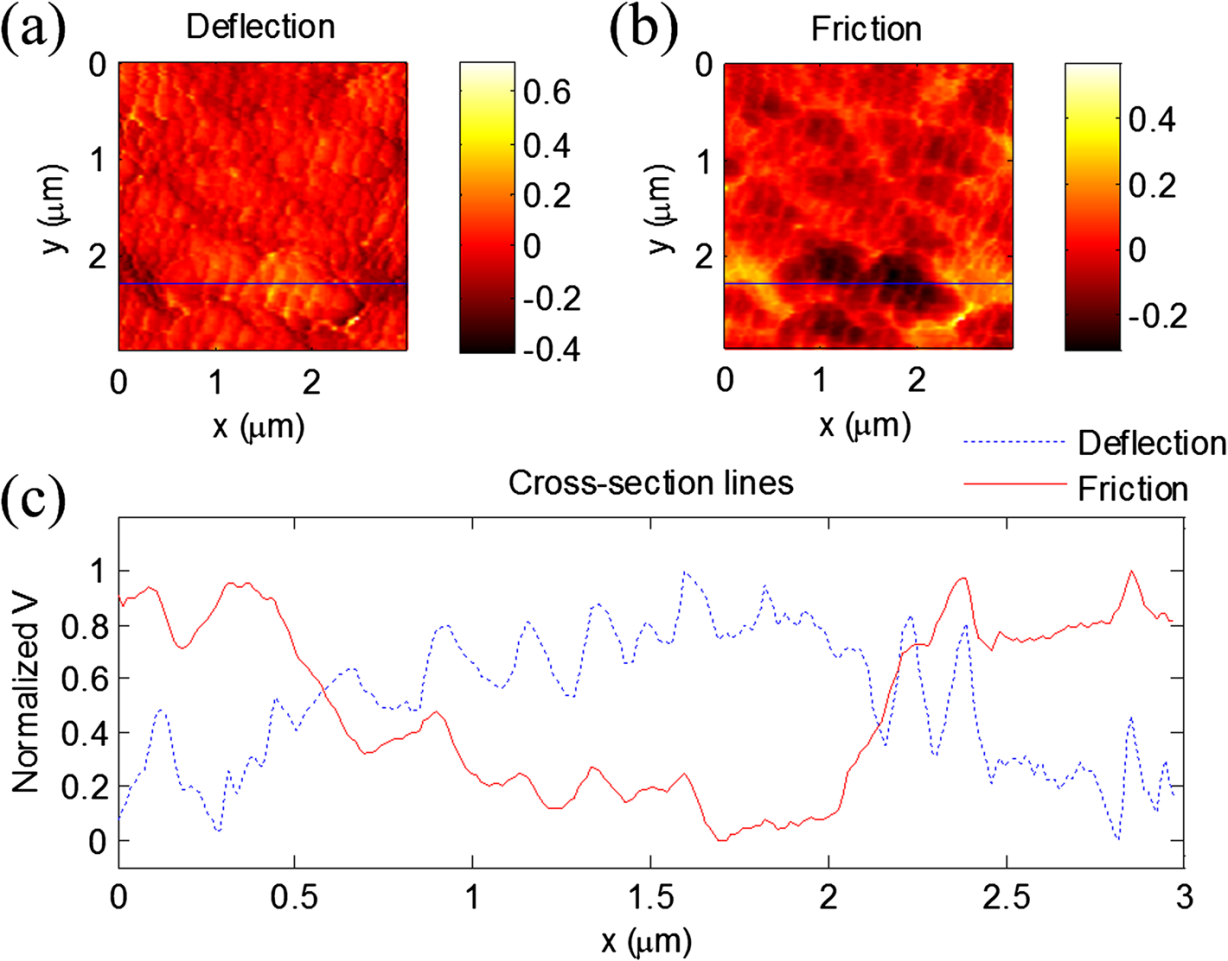
**HSSFM images of the FTO conductive glass surface. (a)** Deflection image, **(b)** friction force image, and **(c)** cross-section lines performed as shown in **(a)** and **(b)**.

On a flat surface, the larger width of the friction retrace-trace curves (the friction loop) means a larger friction force [44]. In our HSSFM, since the average scan speed can easily reach approximately 3 cm/s [42], it would be very useful in the research of friction phenomena at speeds for practical use. So thirdly, the friction force signals from the scan retrace-trace loop corresponding to the forward scan and backward scan on the monocrystalline silicon surface under different normal forces and scan speeds are investigated. In experiments, increasing the normal force was realized by adjusting the set-point value from 0.5 to 1.5 V. So, the maximum force applied on the tip would be three times larger than the minimum force. When the line scan rate was set at approximately 1.5 kHz, increasing the scan speed was carried out by enlarging the scan size from 3 to 5 μm. The corresponding average velocity varies from approximately 0.9 cm/s to approximately 1.5 cm/s. The typical result curves are shown in Figure 4. In Figure 4a, the width of the friction force curves increases while the normal force increases in a general trend. But the friction force curves show fluctuations when the normal force is small. From Figure 4b, the width of the friction force curves increases with the increase in scan speed. The results agree well with existing experimental results on frictional property research with FFM [44,45].Finally, FTO conductive glasses have been studied. Deflection and friction force images are obtained simultaneously, as shown in Figure 5. These images were acquired at 25 FPS, and the scan size is approximately 3 μm × 3 μm. As shown in Figure 5a, the surface of the FTO conductive glass is smooth without obvious macroscopic defects in the scan area, such as cavities or bubbles. The size of the particles on the FTO conductive film estimated from the images is about tens of nanometers. The interesting point is that in the friction force image, as shown in Figure 5b, specific features that are not visible in the deflection image can be seen clearly. One possible explanation is that although the surface is smooth, the material of the conductive oxide film is not uniform in the local area, which might cause the different frictional properties. Figure 5c shows the cross sections of the deflection and friction force signals marked by the lines in Figure 5a,b. The center part of the friction signal also verified that different frictional properties can be distinguished on a nanometer scale.

## Conclusions

In summary, an alternative high-speed scanning force microscopy method based on resonance-type piezoelectric bimorph scanner in the contact mode has been developed. The modified optical beam deflection scheme is suitable for detecting the deformations of small cantilevers and high-speed imaging. The control and data acquisition program has the ability to real-time capture deflection and friction force images simultaneously. High-speed deflection and friction force images of sample surface have been obtained successfully. A series of images of a test grating, a thin gold film, and FTO glass slides were taken with imaging rate at 25 FPS and the average scan speed reached approximately 2.5 cm/s. These results demonstrate that the method combines the advantages of both observing the dynamic processes on sample surface and monitoring the differences in frictional properties on the nanometer scale in real time. It is believe that the method would have many great potential applications, such as real-time surface inspections and nanotribology studies.

## Abbreviations

AFM: atomic force microscopy; AI: analog input; AO: analog output; FFM: friction force microscopy; FPS: frames per second; FTO: fluorine-doped tin oxide; HSAFM: high-speed atomic force microscopy; HSSFM: high-speed scanning force microscopy; OBD: optical beam deflection; PSD: position-sensitive detector.

## Competing interests

The authors declare that they have no competing interests.

## Authors’ contributions

The work presented here was carried out in collaboration among all authors. GS defined the research theme. WC designed the experimental setup and methods. HF and JZ performed the experiments and processed the data. WC and GS drafted the manuscript. All authors read and approved the final manuscript.
